# Global patterns and key drivers of stream nitrogen concentration: A machine learning approach

**DOI:** 10.1016/j.scitotenv.2023.161623

**Published:** 2023-04-10

**Authors:** Razi Sheikholeslami, Jim W. Hall

**Affiliations:** a School of Geography and the Environment, University of Oxford, Oxford, UK; b Environmental Change Institute, University of Oxford, Oxford, UK; c Department of Civil Engineering, Sharif University of Technology, Tehran, Iran

**Keywords:** Nitrogen pollution, Global water quality, Random forests, Hotspot analysis, Variable importance

## Abstract

Anthropogenic loading of nitrogen to river systems can pose serious health hazards and create critical environmental threats. Quantification of the magnitude and impact of freshwater nitrogen requires identifying key controls of nitrogen dynamics and analyzing both the past and present patterns of nitrogen flows. To tackle this challenge, we adopted a machine learning (ML) approach and built an ML-driven representation that captures spatiotemporal variability in nitrogen concentrations at global scale. Our model uses random forests to regress a large sample of monthly measured stream nitrogen concentrations onto a set of 17 predictors with a spatial resolution of 0.5-degree over the 1990–2013, including observations within the pixel and upstream drivers. The model was validated with data from rivers outside the training dataset and was used to predict nitrogen concentrations in 520 major river basins of the world, including many with scarce or no observations. We predicted that the regions with highest median nitrogen concentrations in their rivers (in 2013) were: United States (Mississippi), Pakistan, Bangladesh, India (Indus, Ganges), China (Yellow, Yangtze, Yongding, Huai), and most of Europe (Rhine, Danube, Vistula, Thames, Trent, Severn). Other major hotspots were the river basins of the Sebou (Morroco), Nakdong (South Korea), Kitakami (Japan), and Egypt's Nile Delta. Our analysis showed that the rate of increase in nitrogen concentration between 1990s and 2000s was greatest in rivers located in eastern China, eastern and central parts of Canada, Baltic states, Pakistan, mainland southeast Asia, and south-eastern Australia. Using a new grouped variable importance measure, we also found that temporality (month of the year and cumulative month count) is the most influential predictor, followed by factors representing hydroclimatic conditions, diffuse nutrient emissions from agriculture, and topographic features. Our model can be further applied to assess strategies designed to reduce nitrogen pollution in freshwater bodies at large spatial scales.

## Highlights

• A random forest model was built to predict global, long-term stream nitrogen concentrations (N).
• The 10-fold cross-validation and out-of-sample testing yielded *R*^*2*^ values of 0.90 and 0.71.
• Spatially explicit, global-coverage, monthly estimations of N were produced from 1990 to 2013.
• Increasing gradient of N (over 20 %) was predicted from the 1990s to 2000s at some major river basins.
• Hydroclimatic variables and diffuse nutrient loads from agriculture showed a major effect on N.

## Graphical abstract

**Figure:**
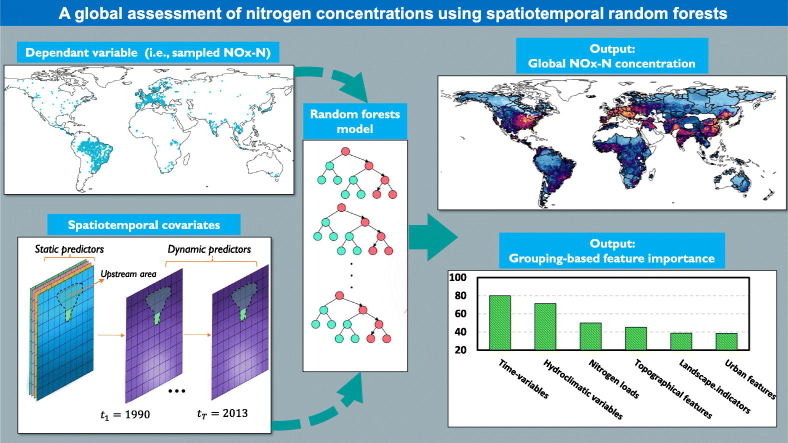

## 1 Introduction

Water quality (WQ) management and pollution control are vital for achieving water security and attaining human wellbeing as reflected in the UN Sustainable Development Goals (SDG6: clean water and sanitation; UNGA, 2015). However, intensifying human activities have imposed immense challenges in sustainable WQ management (van Vliet et al., 2017). During the past century, WQ has declined because of unregulated wastewater discharge and livestock manure and fertilizer draining into catchments and aquifers. In addition, extensive construction of dams, excessive extraction of groundwater, deforestation, and expanding agricultural land use have altered sedimentary processes, mobilization of salts, and nutrient export to river systems, all of which drive WQ deterioration and groundwater pollution in many parts of the world (Scanlon et al., 2007; Seitzinger et al., 2010; Struyf et al., 2010). Furthermore, climate change is expected to have detrimental impacts on WQ due to perturbations of precipitation and temperature patterns in hydrological cycle (Lu et al., 2020; Yang et al., 2019).

In particular, the global nitrogen cycle has been dramatically modified by intensified agricultural practices, with major and widespread impacts upon WQ (Yu et al., 2019). As shown by Green et al. (2004), from 1800 to mid-1990, nitrogen loading to the land surface has increased twofold because of the accelerated anthropogenic activities. For example, the reactive nitrogen produced by humans in 2010 was approximately four times more than the reactive nitrogen created by natural processes (i.e., biological nitrogen fixation) (Fowler et al., 2013; Vitousek et al., 2013). The increased nitrogen flows can be mostly attributed to farming practices. In the past half-century, the global demand for food has boosted the agricultural intensification and expansion, accompanied by the use of fertilizer and animal manure for enhancing crop yields (Foley et al., 2005; Godfray et al., 2010). Zhang et al. (2017) estimated that total manure nitrogen production increased six times during 1860–2014 with an overall significant increasing trend.

Nitrogen is an essential nutrient for growth and nourishment of all living organisms and is a key element of dietary proteins. Nevertheless, an overabundance of nitrogen in water can cause highly undesirable consequences for human health (Miller et al., 2021), such as infant methemoglobinemia (Greer and Shannon, 2005) and colon cancer (McElroy et al., 2008). Excessive nitrogen loading of rivers can also create adverse environmental effects on aquatic and terrestrial ecosystems through three biochemical mechanisms (Jones et al., 2014): eutrophication, acidification, and direct toxicity, which might lead to numerous problems, such as proliferation of harmful algal blooms, exacerbation of hypoxic zones, fish mortality, and loss of biodiversity (Turner et al., 2003; Diaz and Rosenberg, 2008; Clark and Tilman, 2008).

Curbing the aforementioned negative effects of excess levels of nitrogen underscores the need to develop effective WQ management and restoration practices, which often requires mathematical models to assess WQ challenges in future scenarios and appraise possible management responses. There is a large variety of models and modelling concepts in environmental science (Arheimer and Olsson, 2003; Wellen et al., 2015). Importantly, with a growing understanding that WQ problems are global and pervasive, several attempts have been made in the last two decades aimed at introducing and improving large-scale models (Döll et al., 2008; Kauffeldt et al., 2013; Tang et al., 2019; Puy et al., 2022).

The complexity of large-scale models varies based on their objectives and applications. These models broadly fall into two groups: process-based (mechanistic) models and data-driven models. Process-based models historically evolved to incorporate known (basin/local-scale) processes of system behaviors, and their development has mainly focused on solving the conservation equations under certain simplified assumptions (see, e.g., Whitehead et al., 1998; Huang et al., 2009; Raptis et al., 2016). In contrast, data-driven models link WQ parameters (i.e., pollutants) to environmental and anthropogenic variables purely based on given data, without explicit mechanistic information on the processes. Data-driven models are often constructed using either empirical/statistical relationships or machine learning (ML) techniques.

Although process-based models are important tools for understanding physical mechanisms, their robustness and accuracy may suffer from our incomplete knowledge of the hydrogeochemical processes and heterogenous physical properties of catchment systems. These models often have many parameters that need to be calibrated (or estimated), which is sometimes troublesome due to wide ranges of parameters and complex interactions among them. Further, a limited number of observation sites, required to configure (i.e., initialize, parameterize, and calibrate) such models, restricts their usefulness. Another critical limiting factor for the application of these models at large scales is computational demand. Their simulation time typically exceeds the computational resources available for a comprehensive analysis of the model behavior under different conditions.

On the other hand, the opportunity presented by ML-driven models is based on the fact that although observational data for global WQ indicators are scarce, data that measure the drivers of these parameters (i.e., predictors) are not. Another important advantage of modern ML algorithms is their high efficiency during the training procedure. Thus, ML-based models can offer valid and computationally frugal alternatives for projection of future change effects on surface WQ. The recent explosion of large geo-environmental datasets along with rapid progress in artificial intelligence technology have caused ML methods to attain outstanding results in the regression estimation of WQ indicators at a range of spatiotemporal scales (Tiyasha et al., 2020; Sujay Raghavendra and Deka, 2014). ML-based models can efficiently extract patterns from diverse, large, and high-dimensional datasets, possibly including those that are typically not used in process-based models. Notwithstanding the success of ML-based WQ models, a few pitfalls have hampered their wider adoption: first is the lack of interpretability of ML-based models. These models are often built for prediction and do not provide physical relationships, i.e., they are treated as “black boxes”. Second, and more fundamentally, the ML-based estimations are typically prone to uncertainty because of the finite sample size, i.e., not knowing the output variable at unsampled regions outside the training dataset. Third, choosing the best ML algorithm might not be easy due to the existence of many different algorithms.

Table 1 provides an overview of notable studies from the last decade that have employed ML to simulate WQ at large spatial scales. Most of the algorithms in Table 1 are generally based on one of the following two learning concepts: a neuromorphic learning system, such as artificial neural networks (ANN), deep learning (DP), extreme learning machines (ELM), and long short-term memory (LSTM), or a tree-like model of decisions, such as decision trees (DT), random forests (RF) and deep cascade forest (DCF). Other approaches include support vector machine (SVM) and multitask learning (MTL).

**Table 1:** Recent studies published from 2011 to 2022 that applied ML to large-scale WQ modelling.

Reference	ML algorithm	Spatial scale		Temporal scale	
		*Resolution*	*Extent*	*Time step*	*Extent*
He et al. (2011b)	ANN	River basins	Japan	Monthly	1995
Álvarez-Cabria et al. (2016)	RF	River basins	Spain	Annual	2003–2009
Ceccaroni et al. (2018)	DT	–	Wadden Sea	Daily	2003–2015
Collins et al. (2019)	MTL	Lakes	United States	Seasonal	1980–2011
Ross and Stock (2019)	DT	–	Chesapeake Bay	Monthly	1986–2007
Russ et al. (2019)	RF	0.5-degree	Global	Annual	1992–2013
Chen et al. (2020)	DT, RF, DCF	River basins	China	Weekly	2012–2018
Dony (2020)	RF	River basins	Great Lakes	Annual	2000–2016
Mellios et al. (2020)	DT, SVM, RF	Lakes	Europe	Monthly	1980–2009
Shen et al. (2020)	RF	30-arc-second	United States	Seasonal	1994–2018
Arias-Rodriguez et al. (2021)	ELM, SVR	Lakes	Mexico	Daily	2013–2019
Thorslund et al., 2021	RF	River basins	Global	Monthly	1980–2010
Wang et al. (2021)	RF	River basins	Texas Gulf Region	Seasonal	2011
Zhi et al. (2021)	LSTM	River basins	United States	Monthly	1980–2014
Virro et al. (2022)	RF	River basins	Estonia	Annual	2016–2020

From our review of the previous studies on ML-based large-scale WQ models, we make three critical observations:

1) Despite a plethora of powerful ML methods, artificial neural networks (ANN) are the most popular methods for WQ modelling. As reported by Tiyasha et al. (2020), from 2013 to 2019, the average number of published papers that used ANN-based WQ models was 20 paper per year and is still increasing.
2) Unlike the statistical WQ models (e.g., Green et al., 2004; Schultz et al., 2006; Mayorga et al., 2010; McDowell et al., 2020), which are often based on the coefficient estimators, our thorough search of the relevant literature indicated that ML methods have rarely been implemented at global scale.
3) Despite being successful in simulating and predicting WQ at catchment-scale, ML methods have not been utilized to provide spatially explicit (gridded) estimates of WQ indicators. Based on our observation, almost all ML models are lumped in space.

These observations highlight three major research gaps and the need for: (1) broadening the use of ML in WQ modelling beyond the ANN-based methods, (2) implementing ML models at global scale to identify patterns and key drivers of in-stream nitrogen concentration, and (3) providing spatially explicit estimations of stream nutrient concentrations using ML methods, particularly for basins with scarce or no observations. Addressing these gaps will help fully exploit the predictive ability of the advanced ML methods and discover new patterns and relationships in WQ datasets that may not be easily revealed through conventional methods.

The aforementioned research gaps motivated our development of a spatiotemporal global WQ model using random forests technique in the present study. To achieve this, we used more than 53,000 site-level measurements recorded in the United Nations Global Freshwater Quality Database (GEMStat; https://gemstat.org/) and a diverse set of temporally evolving spatial predictors. In particular, the overarching objectives of our study are:

1) To build and validate a random forest-based predictive model for evaluating global stream nitrogen concentration using the existing monitoring data.
2) To characterize the spatial and temporal variability in nitrogen concentration and its hotspot areas across major river basins of the world.
3) To implement a new grouped variable importance measure for identifying key determinants of nitrogen contamination at global scale.

## 2 Material and methods

### 2.1 Data collection and processing

#### 2.1.1 Global nitrate-nitrite measurements

For training our ML-based global model and analyzing its performance, we focused on nitrate-nitrite nitrogen (NOx—N) as the response variable in this study. NOx—N is one of the dominant forms of nitrogen and is very soluble in water, which can significantly deteriorate the quality of surface water. Nitrogen in synthetic fertilizer, manure, and wastewater can be decomposed to ammonia, which is then oxidized to NOx—N and will subsequently enter the groundwater, streams, and lakes, leading to eutrophication, hypoxia, or human health implications. We collected NOx—N monitoring data from the GEMStat repository which was established by the UNEP GEMS Water Programme (Barker et al., 2007). GEMStat (https://gemstat.org/) provides an online, globally harmonized, open-access database for WQ at global, regional, and local scales. It currently contains more than 3.5 million observations for rivers, lakes, reservoirs, wetlands, and groundwater systems from approximately 3000 stations.

**Fig. 1:**
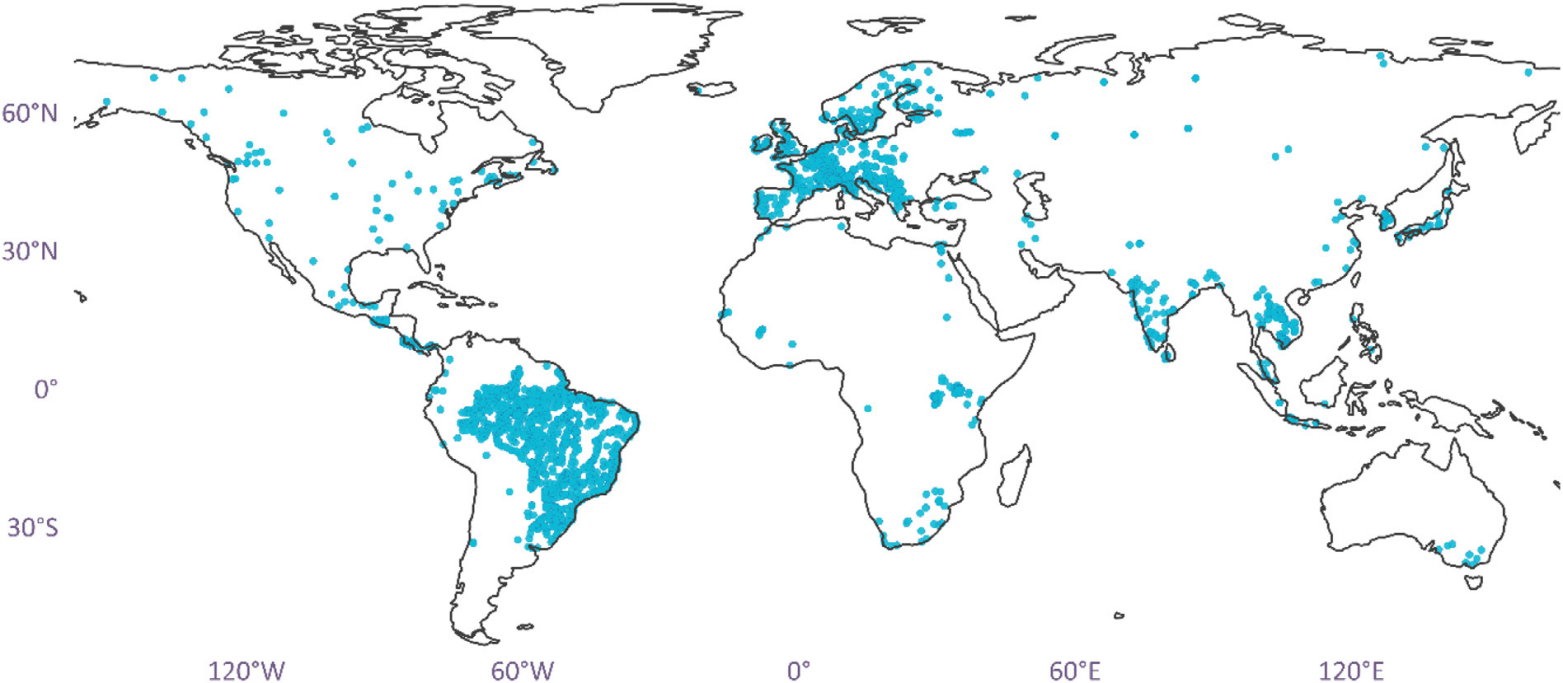
Spatial distribution of GEMStat monitoring stations (blue dots) used in this study for building our ML-driven WQ model. (For interpretation of the references to colour in this figure legend, the reader is referred to the web version of this article.)

NOx—N is well represented in the data repository of GEMStat, with more observations and fewer missing values (da Costa Silva and Dubé, 2013). In total, GEMStat records 82,302 NOx—N observations from 718 stations located in 75 countries (see Fig. 1). For our modelling purpose, we extracted data with the length of 23 years across all river monitoring stations. This resulted in 53,667 temporally consistent NOx—N observations within the 1990–2013 period, from which we computed the monthly aggregated values. The dataset within this temporal range showed a good quality in terms of high temporal consistency and smaller number of missing values. The selected period was also compatible with most of our predictor variable datasets (see Section 2.1.2). Note that the rest of the observations which does not belong to the 1990–2013 period was used for out-of-sample testing (see Section 2.2.4).

**Fig. 2:**
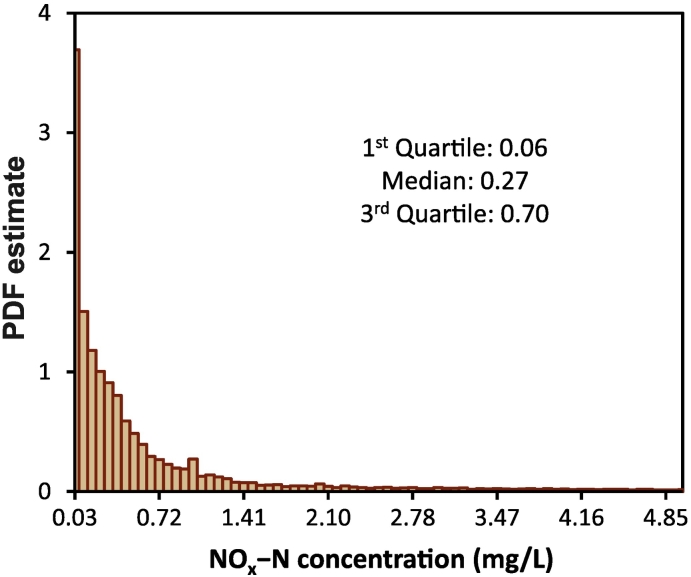
Probability density function (PDF) estimate of the global (monthly aggregated) NOx—N measurements across all GEMStat monitoring stations, over the 1990–2013 period. Note that the sum of the bar areas is less than or equal to 1.

Fig. 2 displays the distribution (PDF) of the global NOx—N values. Note that 10 % of the monthly observations in our training dataset have values smaller than 0.02 mg/L (10th percentile) and 10 % of them have values larger than 2.00 mg/L (90th percentile). Also, about 1.5 % of the GEMStat-monthly NOx—N measurements are less than 0.001 mg/L. The mean and standard deviation of the monthly measurements are 0.78 and 2.80 mg/L, respectively. To improve the data symmetry and suitability for use in our ML model, all observed NOx—N concentrations were transformed using Box–Cox technique. The optimal Box–Cox transformation parameter was obtained using the maximum-likelihood approach.

#### 2.1.2 Predictive variables selection

Several processes interacting at different spatiotemporal scales, along with varying intensities, drive variability of the nitrogen in water. Thus, the primary concern of using ML methods for WQ modelling is selecting an optimal combination of the predictor variables. In this study, we systematically identified predictors considering three selection criteria, including process representation, model complexity, and data availability. We determined a set of variables likely to control nitrogen concentration in a process-informed manner through an extensive literature review (see, e.g., Lintern et al., 2018; Billen et al., 2013; Bouwman et al., 2013; and the references therein) and using domain knowledge obtained from consultation with experienced modelers. Additionally, we chose the set of relevant and discriminative predictors based on the open data criterion, i.e., those datasets which are readily available in existing (online) resources were included in the model. This resulted in a parsimonious, but still comprehensive, collection of the 15 explanatory variables as listed in Table A1 of Supplementary data.

We categorized the selected space-time variables (Table A1) into five groups of primary predictors as follows:

• *Nitrogen loads*: The nitrogen runoff from agriculture, which is often related to both synthetic fertilizer and manure, can increase stream nitrogen levels. The excessive use of fertilizer and manure together with a relatively low nutrient use efficiency by crops have resulted in considerable enrichment of soils with nitrogen, and overland flow from such areas during/after a rainfall event may contain relatively high levels of nitrogen. Thus, diffuse nitrogen export from agriculture, particularly from cropland, has been commonly considered as one of the critical factors degrading WQ. The global gridded data of annual synthetic nitrogen fertilizer use rate (N.fertlz) (Lu and Tian, 2017) and distribution of nitrogen produced in manure (N.manure) (Potter et al., 2010) were included in this group to represent availability of nutrients in runoff from agricultural lands.
• *Landscape indicators*: Deforestation and expanding agricultural land use have been proven to exert a strong influence on stream hydrology and nutrient transport through river systems. Water quality deterioration have been often reported to be positively correlated with agriculture land use, while negatively related to cover types of vegetation. Due to the prominent role of land characteristics on diffuse source pollution, this group contains the fractions of cropland area (Crop.frac) and forest cover (Forest.frac) as the predictor variables. The forest cover fraction was derived from observations and products of the MODIS Sensor aboard EOS-TERRA with annual temporal resolution (Townsend and DiMiceli, 2015). The annual fraction of cropland area was obtained from Jackson et al. (2019).
• *Urban features*: Because of population and economic growth, which leads to changes in diet, fast urbanization, and development of wastewater treatment facilities, urban areas have shown to affect WQ. Particularly, point source inputs from human population in the catchments have been identified as one of the strong predictors for nitrogen exports at highly populated basins. This group accounts for discharges from urban areas and includes population, urban fraction (Urban.area), wastewater production (WWP) and treatment (WWT). Annual population data was obtained from the gridded global datasets for gross domestic product and human development index over 1990–2015 (Kummu et al., 2018). For each grid cell, the fraction of urban area and wastewater production and treatment was derived from Gao and O'Neill (2020) and Jones et al. (2021), respectively.
• *Hydroclimatic variables*: The hydroclimatic conditions were represented by monthly values of precipitation, air temperature, and runoff. These predictors have marked influence on physical processes that govern dynamics of WQ, such as contaminant mobilization, transport, storage, transformation, and dilution. In the present study, terrestrial air temperature and precipitation data were extracted from the 1900–2014 gridded monthly time series produced by Willmott and Matsuura (2001) with the spatial resolution of 0.5-degree. We also used a global gridded monthly reconstruction of runoff data (Ghiggi et al., 2019) to estimate the amount of water drained from a grid cell eventually entering the river system.
• *Topographical features*: There are four variables in this group, including: longitude, latitude, elevation, and upstream area. These factors influence nutrient transport into and within the stream network because topography is a major factor controlling the volume, velocity, and direction of flow in the catchment. We used DEM dataset with 15-arc-second spatial resolutions provided by the USGS (Danielson and Gesch, 2011) as the source data for extracting topographical features.

The catchment characteristics upstream of the monitoring site significantly influence the quality of water at each sampling point. Therefore, the predictors for instream nitrogen concentration prediction not only should cover the properties within the grid cell of interest but also should consider information from the upstream grid cells. Previous studies have identified that the extent of human activities (i.e., agriculture and urbanization), nutrients, and hydroclimatic conditions in a catchment are among the key upstream drivers affecting spatial variability in WQ (Lintern et al., 2018). Hence, we defined two distinct set of variables in this study: upstream drivers and local drivers (i.e., observations within the pixel). For each monitoring site, the upstream drivers were estimated for nitrogen loads, landscape indicators, urban areas, and hydroclimatic variables (see Section 2.1.3) and the rest of the predictors were considered as local.

#### 2.1.3 Data preparation

Since major drainage basins have historically played a vital role in the localization of cities as well as the distribution of human activities worldwide, our temporal and spatial analysis was centered on the world's 520 major river basins. In this paper, the basin polygons (see Fig. A1 in Supplementary data) were derived from the Global Runoff Data Centre (GRDC, 2020). The total area of these basins covers ∼70 % of the global land surface area with ∼60 % of the global population; therefore, the quality of water in these basins is crucial for millions of their inhabitants. The selected basins represent a wide range of climatology, such as tropical, humid, semi-arid, and arid, and cover a wide range of hydrological regimes, such as snowmelt-fed regime with high summer flows to rainfed regime supplied by winter-to-spring precipitations. There is also a large variability in the size – the smallest basin – Coatán catchment located in the Southeast of Chiapas state of Mexico (∼700 km^2^) to the largest – the Amazon River basin (∼5,000,000 km^2^).

To account for upstream-downstream linkages, the number of grid cells (or area) which lie upslope of each cell, and which are draining into that downslope cell was determined using the DEM data and D8 flow accumulation algorithm of the ArcGIS's catchment delineation tool. This enabled us to calculate the accumulated values of the nitrogen loads, percentage of cropland, forest cover fraction, urban areas, and hydroclimatic variables for the area upstream from each grid cell. These upstream characteristics were used in training our ML-driven model and helped us better represent the impacts of upstream events and activities on WQ in the near and far downstream. Particularly for rivers in large basins (e.g., Mississippi, Amazon, Mekong, etc.) that are running across many grid cells, it is important to incorporate the effect of upstream (management/catchment) characteristics into the model.

Moreover, all coordinates for the selected predictor variables were projected into the coordinate reference system (CRS). Then, for each predictor, the *k*-nearest neighbour imputation was applied to replace the missing values with the mean values obtained from the 100 nearest neighbours found in the data via a Euclidean distance metric. Because of the different resolutions, we subsequently reshaped all predictors to the same spatial resolution of 0.5-degree using bilinear interpolation resampling technique and the same temporal interval. After standardizing data (i.e., centering at the mean and scaling by the standard deviation), all predictor variables were Box-Cox transformed. Finally, these predictors were matched to the monthly NOx—N observations of monitoring stations. This resulted in a training dataset containing a total of 42,794 matched monthly measurements and all predictors. In this way, we produced a consistent, spatially explicit global-coverage datasets for the years 1990–2013.

### 2.2 Model development

#### 2.2.1 The standard random forest model

Random forest is a relatively modern ML method that basically uses the assemblage of multiple iterations of decision trees. With the capability of processing large environmental datasets and handling nonlinear relationships, random forest has been increasingly become a popular data analysis method that outperforms other ML-based tools (Fox et al., 2020). There has been extensive theoretical exploration and testing of random forests against other data-driven methods in WQ modelling. By way of example, Chen et al. (2020) compared water quality prediction performance of several learning models, including logistic regression, linear discriminant analysis, support vector machine, decision tree, and random forest across major rivers and lakes in China. Also, performance of the random forest algorithm in simulating groundwater nitrate contamination at the African continent scale was evaluated in comparison to the multiple linear regression by Ouedraogo et al. (2019). Details of random forest algorithm can be found in Breiman (2001); Friedman et al. (2001) and were briefly described below.

Random forest is a nonparametric regression technique which contains a collection, or “forest”, of independent regression trees {*t*^(*k*)^ : *k* = 1, 2, …, *B*} as base learners. Each growing tree, *t*^(*k*)^, in the forest is made from bootstrap samples drawn, with replacement, from the original training dataset. These trees are formed by randomly selecting *m* variables out of *p* predictors at each parent node, and the best split-point is found among these *m* variables using a greedy recursive algorithm to create two child nodes. This greedy algorithm recursively partitions a group of *m* predictor variables based on identifying the predictor that minimizes error when regressed against the output of interest. Note that trees are grown deep with no pruning.

In the random forests algorithm, the remaining observations, which are not included in the bootstrap sample, are called Out-Of-Bag (OOB) sample (also referred to as test set). For each tree, the prediction performance (measured, for example, by mean squared error (MSE)) on the OOB sample is recorded and is used for measuring the prediction error of the *k*-th regression tree. After calculating all individual tree predictions, they are averaged to obtain the final random forests prediction. This process works as a cross-validation for each tree in the forest and provides an unbiased overall model error estimate (Prasad et al., 2006). Hence, the prediction at a new site, ${\hat{f}}_{\varphi }$, with predictor vector *x*, is found by estimating the mean value of all regression trees, ${\hat{f}}^{\left(k\right)}$, i.e.,

(1) $${\hat{f}}_{\varphi }\left(x\right)=\frac{1}{B}\sum_{k=1}^{B}{\hat{f}}^{\left(k\right)}\left(x;\theta_{k}\right)$$

where the variable *θ*_*k*_ determines which predictors get included in the *k*-th tree. Eq. (1) explains the main idea behind random forests, which is averaging over *B* fitted regression trees to reduce variance, and thus to improve predictive performance compared to a single regression tree.

#### 2.2.2 A grouping-based algorithm for measuring variable importance

An important inherent feature of the random forest is its ability to assess the variables' predicting strength (expressed as variable importance ranking) using the recorded OOB prediction errors. This feature helps screen the relatively small number of important factors from the pool of selected predictor variables, thereby identifying which variables are strongly driving the output of interest. Random forest evaluates variable importance by estimating the mean decrease in prediction accuracy before and after randomly permuting the values of a given predictor in the OOB data. For the *k*-th tree, when randomly permuting the *i*-th predictor, MSE of the OOB data can be calculated as (Wei et al., 2015):

(2) $${\mathrm{MSE}}^{\left(k\right)}=\frac{1}{N_{\mathrm{OOB}}}\sum_{j=1}^{N_{\mathrm{OOB}}}\left({y_{j}}^{\left(k\right)}-{{\hat{f}}_{j}}^{\left(k\right)}\right)\;\text{and}\;{{\mathrm{MSE}}_{i}}^{\left(k\right)}=\frac{1}{N_{\mathrm{OOB}}}\sum_{j=1}^{N_{\mathrm{OOB}}}\left({y_{j}}^{\left(k\right)}-{{\hat{f}}_{j,i}}^{\left(k\right)}\right)$$

where *N*_*OOB*_ is the OOB sample size; ${y_{j}}^{\left(k\right)}$ is the *j*-th observation in the OOB data of the *k*-th tree; and ${{\hat{f}}_{j}}^{\left(k\right)}$ and ${{\hat{f}}_{j,i}}^{\left(k\right)}$ are predictions of the OOB data before and after randomly permuting *i*-th predictor, respectively. Note that, in Eq. (2), if the *i*-th predictor is not selected on the split-point of any node of the *k*-th tree, then then ${{\hat{f}}_{j}}^{\left(k\right)}={{\hat{f}}_{j,i}}^{\left(k\right)}$ (for all *j*), and thus ${\mathrm{MSE}}^{\left(k\right)}={{\mathrm{MSE}}_{i}}^{\left(k\right)}$.

Assuming that permuting the values of one predictor cannot increase prediction errors, if that predictor dose not significantly impact model accuracy, the difference between *MSE*^(*k*)^ and ${{\mathrm{MSE}}_{i}}^{\left(k\right)}$ is then averaged over all trees and considered as an importance measure. In other words, the overall importance of *i*-th predictor, *PIM*_*i*_, can be expressed as the mean decrease in accuracy values of all trees:

(3) $${\mathrm{PIM}}_{i}=\frac{1}{B}\sum_{k=1}^{B}\left({{\mathrm{MSE}}_{i}}^{\left(k\right)}-{\mathrm{MSE}}^{\left(k\right)}\right)$$

While knowing the individual importance of predictors is useful, we might wish to know which groups of predictors are important given a pre-defined grouping. Also, it is often the case that multiple predictors may be almost equally important or there may be collinearity between predictors, and thus we are interested not only in which predictors are important but also in their joint effect or importance. In addition, for high-dimensional settings with many variables, analyzing the influence of individual variables visually might be troublesome and result in an information abundance. Such needs warrant the development and application of an importance measure that can work with pre-specified groups of factors for which permutation importance can be estimated.

Therefore, we used an alternative permutation importance measure for groups of variables. Our strategy is based on Gregorutti et al. (2015); Au et al. (2022) and starts by putting a pre-defined subset of predictors into some meta-features. These feature groups can be specified based on variables' relationship with each other or a priori knowledge. For example, in our application, we grouped the predictors into five classes, i.e., nitrogen load, landscape indicators, urban features, hydroclimatic variables, and topographic features. Then, unlike the traditional feature importance algorithm, for each meta-feature all variables associated with that group will be randomly permuted together and the increase of the prediction error of the model is computed. After permuting all predictors in all meta-features, we can calculate the grouped variable importance by comparing the overall decrease in accuracy of the regressor (see Eqs. (2), (3)). The worse the model performs when a group is randomized, the more important that group is in terms of predicting the response variable. Note that this grouping-based importance measure permutes individual groups of predictors from the total set of predictors, and accordingly the obtained importance score accounts for interactions within the groups but ignores any interactions with other predictors outside the considered group.

#### 2.2.3 Incorporating space and time into the standard random forest

The standard random forest method does not exploit the spatial and temporal information of the observations, essentially being ‘*aspatial*’ and ‘*nontemporal*’ algorithm. When used for modelling spatiotemporal data, the standard method generates a single output which is estimated from the whole extent of the study area, using all available data points over time. For WQ modelling, however, this can be a crucial problem because WQ constituents are naturally characterized by spatial and temporal heterogeneity, which indicates that the true underlying relationship between predictant and predictors can be spatially and temporally varying. Therefore, it is necessary to construct a random forest model that can capture the spatial-temporal characteristics of the nitrogen levels.

Generally, two strategies have been proposed in the literature to account for spatial information. The first, and more sophisticated, strategy uses a hybrid modelling framework by embedding Kriging and Gaussian process modelling into the standard random forests to model spatial correlation structure of data (see, e.g., Saha et al., 2021; Canion et al., 2019). The second strategy is more straightforward, which explicitly utilizes geographic information as additional inputs, for example, by adding geographic coordinates (Behrens et al., 2018; Meng et al., 2018) or other spatial distances (Li et al., 2011; Wei et al., 2019) into the list of predictors. We followed the second strategy and incorporated the latitude (lat) and longitude (lon) of each grid cell into our random forest model as two additional predictors since they contain geographical information.

Regarding the time dimension, we relied on a commonly used approach introduced by Cohn et al. (1989) (also known as L7 model), where the constituent concentration is related to three explanatory variables: discharge, time, and season. Similar approach was implemented in some regression-based WQ models and it has been shown that this scheme can reasonably explain temporal variability in constituent concentrations (Hirsch et al., 2010; McDowell et al., 2021). Therefore, we added two new auxiliary inputs, namely Cumulative Month since the beginning of the simulation (CM) and Month of the Year (MOY) to represent distance in the time domain, thereby better capturing dynamics of nitrogen levels. These variables were included in one feature group, which has been named as time-variables.

#### 2.2.4 Model training and tuning

We produced a global space-time regression matrix for our model by binding two time-variables and values of the 15 predictors introduced in Section 2.1.2 (see Table A1) together. As a result, the final set of predictors consists of 17 covariates which are expected to be key determinants of source, mobilization, or delivery of nitrogen concentrations globally. Fitting a random forest model for this space-time regression matrix follows the same procedure as described previously in Section 2.2.1. Regarding the random forest parameter tuning, note that, unlike many data-driven algorithms, there are only two tuning parameters need to be calibrated: (1) the number of variables selected randomly at each node (*m*) and (2) the number of trees in the forest (*B*). For a random forest model, *B* controls the variation among different regression trees and *m* determines the extent of overfitting. Increasing the number of trees typically decreases the prediction error of the random forest up to a certain point. For *B* values larger than this threshold, model accuracy changes very slightly, whereas computational demand increases significantly (Freeman et al., 2016). Here, we optimized these parameters by trying different combinations of *B* and *m* and evaluating the performance of each model. Finally, based on the best fitted model, we generated timeseries of NOx—N concentrations as raster maps within the space-time domain of interest.

When training ML methods, the performance of the algorithm is highly sensitive to how the dataset is partitioned into training and testing samples. To tackle this issue, we implemented *k*-fold cross validation strategy. This validation strategy starts by randomly splitting dataset into *k* subsets of similar size. Then, a random forest is learned using observations in *k* − 1 subsets, and an error value is calculated by testing the algorithm on the remaining set. The *k*-fold cross validation estimation of the error is the average value of the errors committed in each fold. Additionally, the process of partitioning can be repeated several times (known as repeated *k*-fold cross validation) to create multiple random splits of the dataset. It has been often presumed that performing repeated *k*-fold cross validation on different random partitions can stabilize the error estimation (Rodriguez et al., 2010). In the present work, we conducted 10-fold cross validation with 3 repeats. The overall cross validation accuracy was taken as the average of mean absolute error (MAE) obtained from each repeat. MAE is a simple and easily interpretable measure of error on the same scale as observed values.

Furthermore, we carried out an out-of-sample validation, where a portion of the data, which was not used to build the model, served as an independent test set for assessing how well the model generalize to new data. Recall that, to train the random forest model, we used monthly NOx—N observations obtained from GEMStat, which cover 1990 to 2013 (sample size = 42,794). We considered the rest of the observations (sample size = 28,802), which does not belong to this temporal duration, as *unseen* data for out-of-sample testing. This out-of-sample validation strategy helped us detect possible overfitting issues in our model.

## 3 Results

### 3.1 Model fitting

**Fig. 3:**
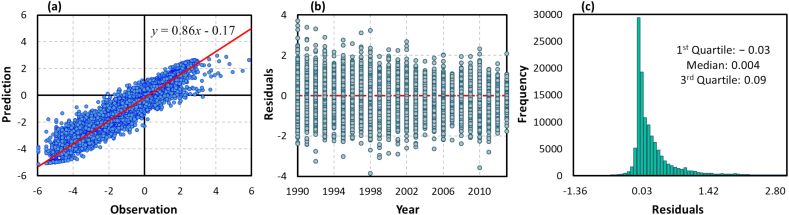
Performance of the random forest model for predicting NOx—N concentrations. Subplot (a) shows the pairwise scatter plot of the monthly average observations versus corresponding cross-validated predictions. The solid red line represents the linear regression fit. Subplot (b) shows temporal distribution (annual average) of the residuals and subplot (c) depicts histogram of the residuals, obtained from all training stations. Note that subplots (a) and (b) are in Box–Cox transformed space, whereas subplot (c) is in the original scale (back transformed). (For interpretation of the references to colour in this figure legend, the reader is referred to the web version of this article.)

The global random forest model was tuned by varying *B* between 200, 500, and 1000, and *m* between 2, 4, 6, and 8. Based on these experiments, the optimal setting was found to be *B* = 1000 and *m* = 4. For the final model, the 10-fold cross-validation (repeated 3 times) yielded average MAE value of 0.42, suggesting a good performance of the model. As additional performance criteria, we calculated the coefficient of determination (*R*^2^) and root-mean-square deviation (RMSD) between all cross-validated predictions and their corresponding observations to diagnose the variation in the errors in a set of predictions. Fig. 3(a) further verifies the effectiveness of the model by comparing response values estimated by the trained model with the observed values (in a Box-Cox transformed scale), across all monitoring sites. The observations lie reasonably close to the predicted concentrations with RMSD = 0.45. The obtained *R*^2^ value implies that the proposed random forest model has accounted for 90 % of the variability in the NOx—N values.

We also performed a thorough residual analysis in Fig. 3. The temporal trend of residuals for all monitoring stations (in Box-Cox transformed scale) are displayed in Fig. 3(b). As can be seen, the average annual residuals are approximately cantered around zero and are relatively constant over time and space. This confirms that our model can capture the spatiotemporal structure in the NOx—N data. Fig. 3(c) depicts the histogram of the residuals obtained from all simulated and observed (back transformed) data, which has mean of 0.12 mg/L and standard deviation of 2.40 mg/L. Note that, for poorly predicted NOx—N values, the observations are, in general, larger than predicted values, indicating conservative estimations by the proposed model.

### 3.2 Validation with unseen data

**Fig. 4:**
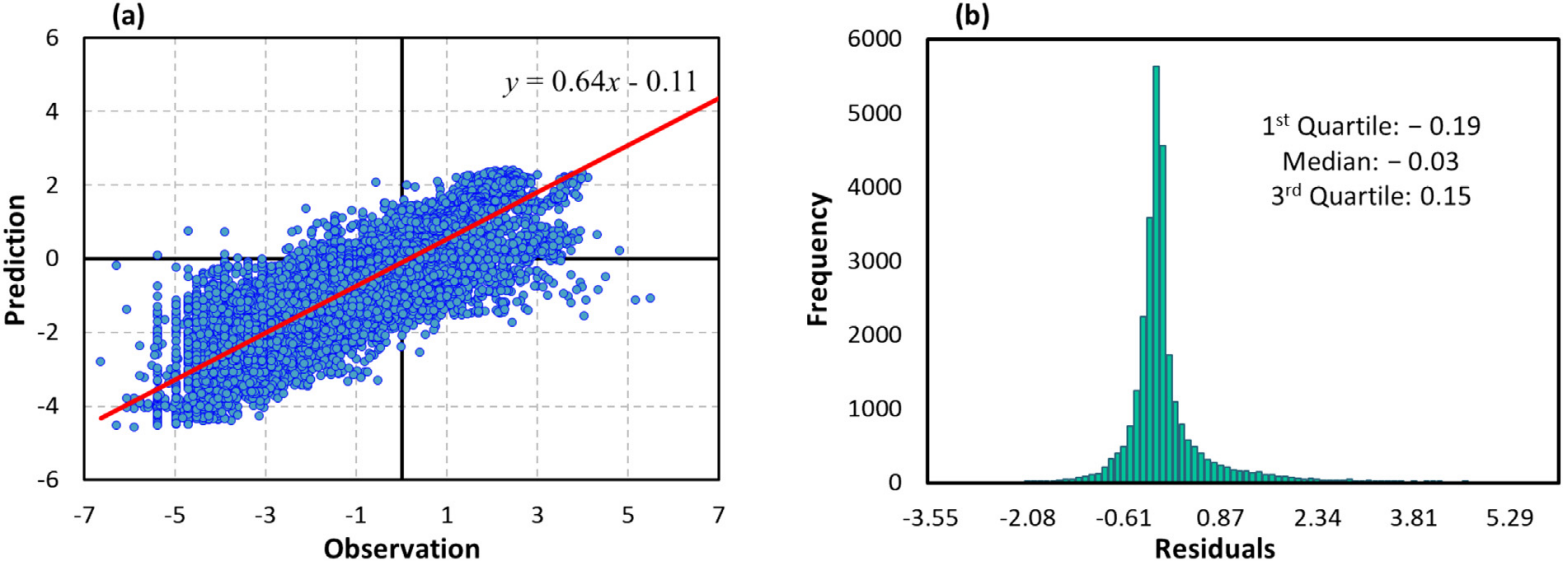
Out-of-sample performance estimation of the random forest model for predicting unseen NOx—N values. Subplot (a) shows the pairwise scatter plot of the monthly average observations versus corresponding predictions in out-of-sample set. The solid red line represents the linear regression fit. Subplot (b) depicts histogram of the residuals, obtained from all out-of-sample data. Note that subplot (a) is in Box–Cox transformed space, while subplot (b) is in the original scale (back transformed). (For interpretation of the references to colour in this figure legend, the reader is referred to the web version of this article.)

Although our results confirm significant evidence of in-sample predictability as evident in Fig. 3, it is necessary to evaluate the out-of-sample predictability as well. Thus, we tested our model on an independent subset of NOx—N observations not seen during the training phase. Fig. 4 shows the out-of-sample validation results for NOx—N estimates.

From the scatterplot in Fig. 4(a), we see that the out-of-sample monthly NOx—N estimates are well correlated with ground measurements and are close to a 1:1 ratio, with MAE and RMSD values of 0.61 and 0.93, respectively, which are higher than those obtained for in-sample test. The obtained *R*^2^ indicates that our model can only explain 71 % of the variation in out-of-sample observations, which is 21 % lower than the in-sample fit. Furthermore, the histogram of the residuals obtained from all simulated and observed out-of-sample data is shown in Fig. 4(b), with mean of 0.31 mg/L and standard deviation of 3.98 mg/L. We observe that the distribution of the residuals is close to being approximately normally distributed, though it has heavier tail than a normal distribution. These results attest out-of-sample validity of the proposed model, and hence it can reproduce the unseen data with reasonable accuracy. The achieved agreement between random forest predictions and independent NOx—N observations provides confidence to the overall approach.

### 3.3 Patterns of nitrogen concentrations and global hotspots

**Fig. 5:**
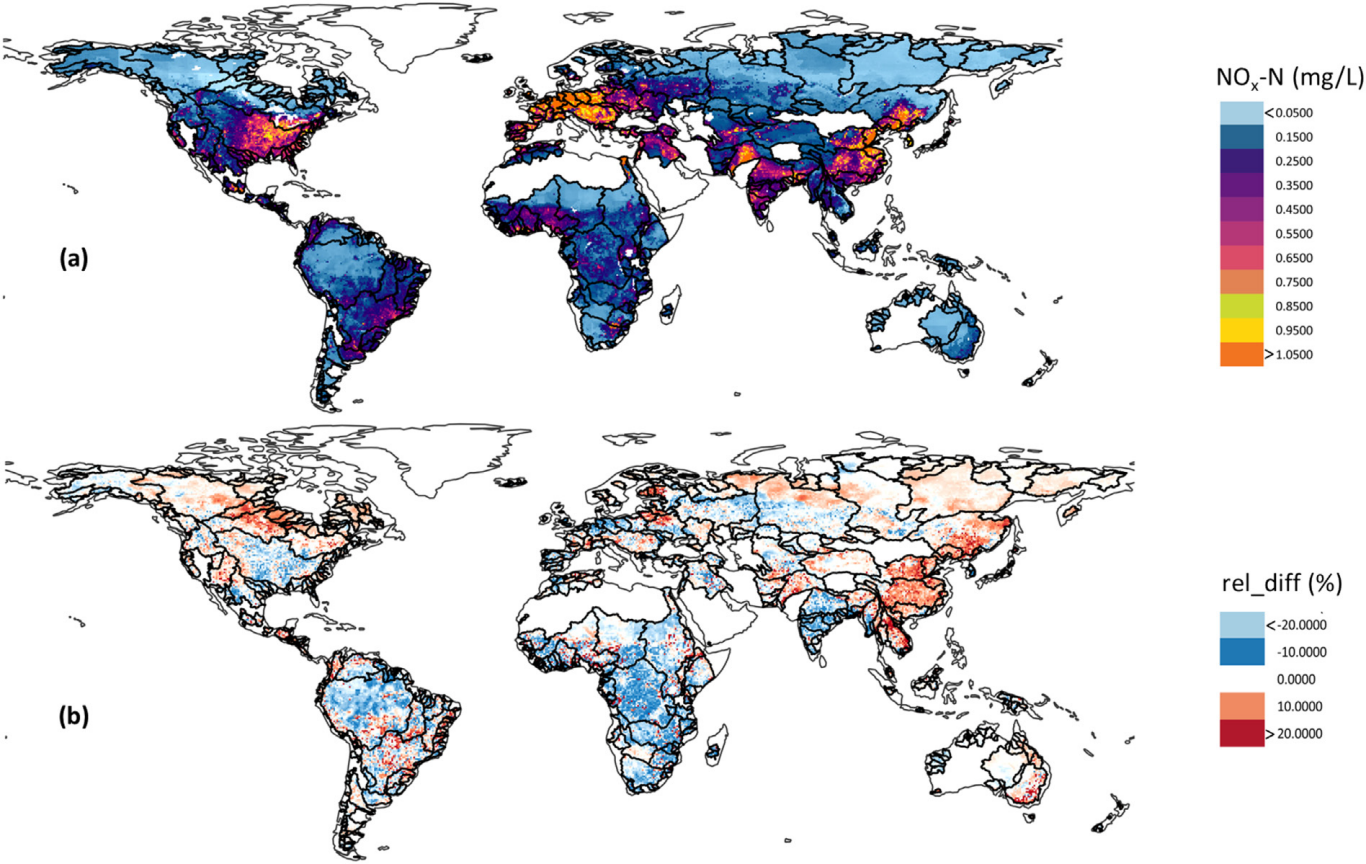
Random forests-based prediction of the spatiotemporal variability in NOx—N concentrations, across major river basins of the world. Subplot (a) shows the simulated global map of NOx—N values averaged over 1990–2013. Subplot (b) shows spatial pattern of the decadal NOx—N changes from the 1990s (1990–1999) to the recent decade (2000−2013).

To characterize global variations of nitrogen, we generated spatial maps for each month using our random forest model for every year in the period of 1990–2013. Fig. 5a (top panel) presents the estimated spatial distributions of mean NOx—N across 520 major river basins averaged over 1990–2013. As can be seen, the estimated NOx—N concentrations exhibit a considerable spatial variability over the globe. The highest rates of nitrogen concentration can be especially found in many European basins, United States, parts of Mexico, southern Brazil, eastern Argentina, West Africa, South Asia, and eastern China. In general, our model predicted higher NOx—N concentrations for densely populated basins with high GDP, such as Rhine and Thames River basins, and basins with extensive agricultural activities, such as Yangtze and Ganges River basins. Other areas with high nitrogen level were predicted in New Zealand, Japan, and parts of South Korea. Our findings generally show substantial consistency with most prior works which used different modelling approaches. For example, a similar spatial pattern, but with few distinctions, was also identified by He et al. (2011a), who estimated global NOx—N concentrations in 1995 using a process-based terrestrial nitrogen cycle model.

Moreover, we clearly observe that the NOx—N concentrations were low for areas with low temperature and little precipitation. The dominant regions of the NOx—N can be found in the Northern Hemisphere, where maximum values occurred in 20°N–60°N (see Fig. A2 in Supplementary data). These latitudes mostly correspond to high agricultural activity and high livestock densities (Potter et al., 2010). In contrast, there are basins with low fertilizer use but high NOx—N concentration. Examples include downstream region of the Amazon River and the midstream region of Congo River. In the Southern Hemisphere, the concentrations are generally much lower, except for 20°S–40°S. In addition, a relatively similar nitrogen variability pattern was predicted by the random forest model for most of the basins in Eurasia and North America, i.e., in high-latitude Northern Hemisphere. This might be partially attributed to the similarities in vegetation, land-use, and hydroclimatic characteristics of sub-Arctic zone. However, more research is needed to explore the underlying biogeochemical mechanisms in these basins.

We also investigated the decadal change in the NOx—N concentration within each grid from the year 1990 through 2013. To do this, we averaged the estimated NOx—N concentration in each grid cell during the 1990s (1990–1999) and 2000s (2000–2013). Fig. 5b (bottom panel) depicts the relative percentage change between these two periods. This map reveals very interesting patterns and can reflect the spatial heterogeneity of the decadal NOx—N change, avoiding extreme values in a specific year.

As shown in Fig. 5b, almost entire eastern China has experienced an enhancement of NOx—N concentration. In addition, eastern and central parts of Canada, parts of South America, southern France, Switzerland, parts of Balkans, Belarus, Baltic states (Latvia, Lithuania, and Estonia), southern Finland, Pakistan, Afghanistan, parts of Russia, mainland southeast Asia (Cambodia, Laos, Burma, Thailand, and Vietnam), and south-eastern Australia showed a significant increasing gradient of the NOx—N in their rivers from 1990s to 2000s (over 20 % at some locations). In opposite to this general increase of nitrogen levels over time, model results also indicate that in some regions of the world there has been a considerable decline in nitrogen levels (at some locations more than 20 % decrease) during the past decades, including south Korea, India, Ukraine, Poland, Germany, United Kingdom, Central Africa, and northern Brazil. This WQ improvement might be due to several factors, for example, the influence of regulations on fertilizer application and potential source reduction, or the enhanced efficiency of treatment plants.

By combining these results, we identified several river basins over the globe where NOx—N was densely concentrated, and accordingly the nitrogen pollution might be most serious. Overall, major hotspots of NOx—N were the river basins of the Mississippi, Sebou, Egypt's Nile Delta, Indus, Ganges, Yellow, Yangtze, Yongding, Huai, Nakdong, Kitakami, Lower Amur, and the Lake Urmia basin as well as most of the European river basins, such as Rhine, Danube, Vistula, Thames, Trent, and Severn.

### 3.4 High importance factors influencing predictions of nitrogen levels

**Fig. 6:**
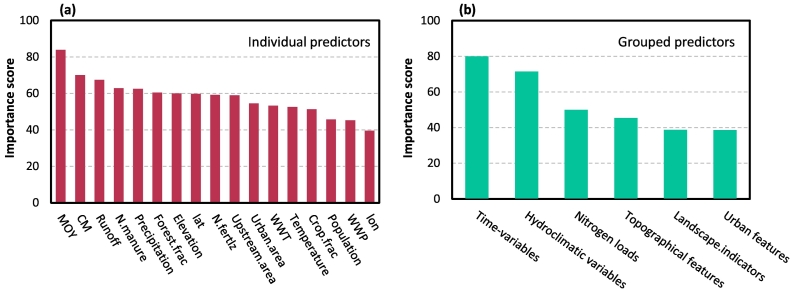
Importance plots derived from the proposed random forest model for (a) individual predictors and (b) grouped predictors. The horizontal axis lists predictor variables in order of decreasing importance. The vertical axis (unitless) represents the mean decrease in accuracy after permuting the variable(s) of interest. Note that only the relative importance scores between predictors should be interpreted, not the absolute values of the scores on y-axis.

We examined predictor variables importances in Fig. 6. The individual importance results in Fig. 6(a) shows that two variables representing temporal dynamics of nitrogen (i.e., MOY and CM) are the most influential variables. This reveals that, distinctly, the most important factor for predicting monthly NOx—N concentrations is time, i.e., cumulative and/or month of the year. In other words, incorporating these variables into the random forest allows our model to fit different spatial patterns for each month of the year. The spatial (location) and time (decades) factors were reported to be among the most significant factors influencing other WQ parameters as well, such as metal concentrations (Dendievel et al., 2022). In Fig. 6(a), other strongly influential predictors are (in rank order): runoff, nitrogen in manure, precipitation, forest cover fraction, and elevation. These major controls of nitrogen levels identified by the random forest model were also reported previously, see, e.g., Shindo et al., 2003; He et al., 2011a; etc.

To characterize the dominant processes affecting model responses, we also implemented the grouping-based importance ranking as described in Section 2.2.2, which ranks predictors according to importance group rather than individually. According to the grouping-based importance results in Fig. 6(b), we can categorize predictor groups into three importance classes: {Time-variables, Hydroclimatic variables} is the strongly influential class; {Nitrogen loads, Topographical features} is the moderately influential class; and {Landscape indicators, Urban features} with least influence on model predictions.

**Fig. 7:**
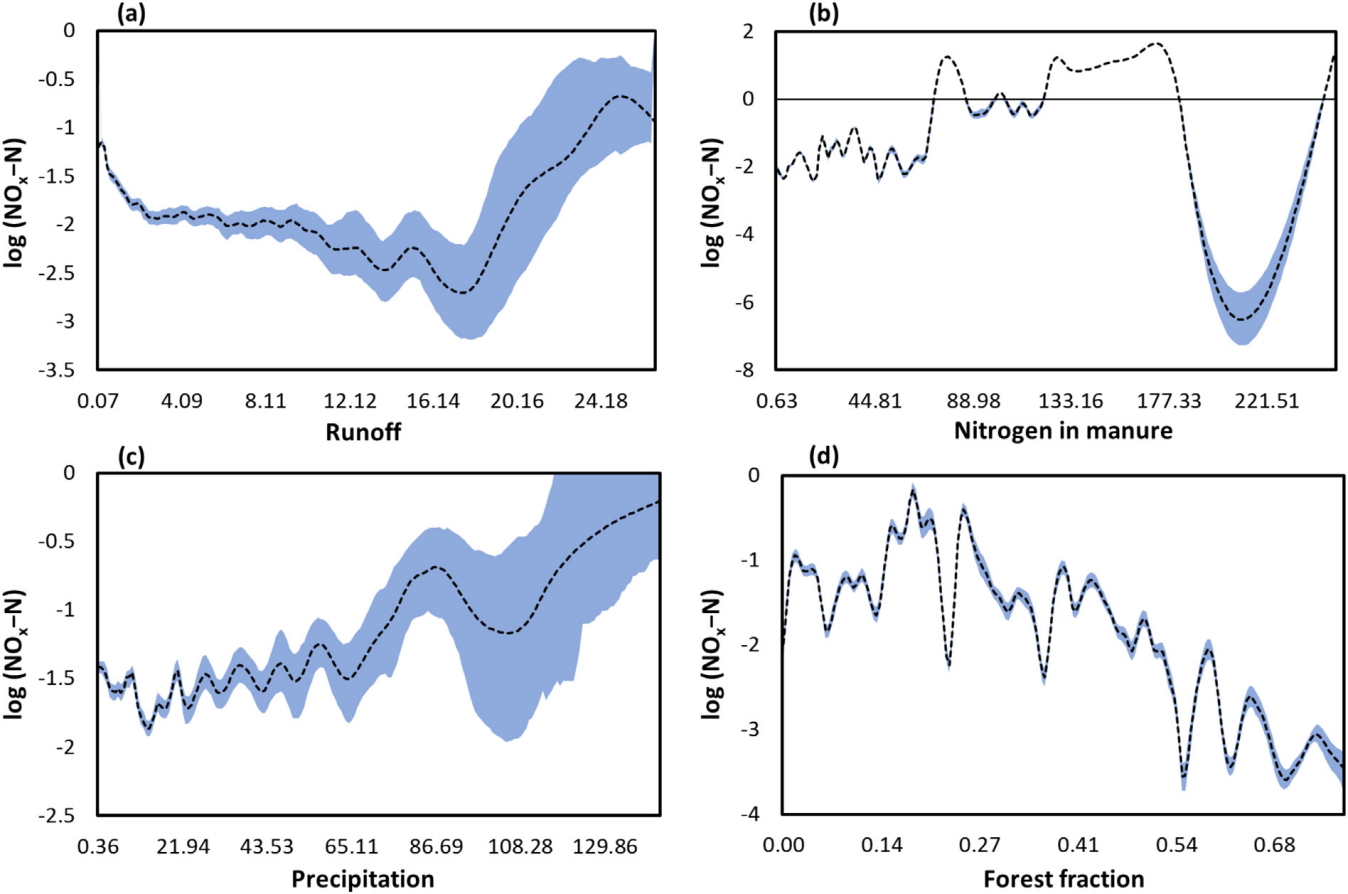
Marginal response curves for the most important predictor variables: (a) runoff, (b) nitrogen in manure, (c) precipitation, and (d) forest cover fraction. A smoothed estimate of the data (dashed lines) with 95 % bootstrapped confidence intervals (shaded areas) were obtained from logarithmic NOx—N values across all monitoring stations, over the 1990–2013 period. We used smoothing spline function and bootstrapping technique for estimating functional relationships between predictors and response variable.

It is also interesting to investigate the individual relationships between predictors and response variable. To this end, we further analyzed how the predicted NOx—N concentrations change by varying the four most important variables. Fig. 7 illustrates the marginal response curves for runoff, nitrogen in manure, precipitation, and the fraction of forest cover. The trend lines (in Fig. 7) were obtained by fitting smoothing spline to logarithm of predicted NOx—N for all monitoring stations during the study period. The magnitude of the 95 % confidence intervals was estimated through bootstrapping.

As can be seen from Fig. 7, there are highly non-linear relationships between all important variables and NOx—N values. Fig. 7(a) reveals that logarithm of NOx—N decreases gradually with increasing runoff to a certain value, then the trend is reversed by a steep increase. However, the relationship between nitrogen in manure and NOx—N is much more complex in Fig. 7(b). These two predictors have momentous effects on predicted nitrogen level as compared to other variables. Furthermore, precipitation seems to have a positive relationship with NOx—N (Fig. 7(c)). It is generally hypothesized that more extreme precipitation events generate greater nutrient runoff (Lu et al., 2020) leading to large contributions of nitrogen from uplands to receiving streams and lakes. On the other hand, the fraction of forest areas in catchments shows, on average, an opposite relationship with NOx—N (Fig. 7(d)) mainly because forests in catchments limit nitrogen delivery to streams. Particularly, riparian forests can mediate non-point source pollution in agricultural areas via intercepting a considerable amount of nitrogen moving towards the stream from uplands.

## 4 Discussion

### 4.1 Comparison with other studies

Our proposed model offers two notable advantages. First, it is a data-driven approach. As such, it estimates nitrogen concentration directly from data by exploiting the random forest's ability of finding complex spatiotemporal patterns (without providing explicit form of them). This makes our model easier to construct in comparison to process-based models. Second, unlike process-based models that are computationally expensive in nature and often suffer from over-parameterization and calibration issues, our model is computationally efficient and is less sensitive to different parameter settings, so it can function quickly over large datasets.

The predictive ability of the proposed model was also compared with other data-driven modelling approaches that attempted to simulate nitrogen concentrations at a global scale. For example, Damania et al. (2019) developed a random forest model to simulate GEMStat's NOx—N observations from 1992 to 2010 across the world and yielded *R*^2^ value of 0.94, which is slightly better than our model (*R*^2^ = 0.90). However, the validity of their model might be questionable (and cannot be verified) because only a single train-test split was implemented in Damania et al. (2019), whereas we conducted the 10-fold cross-validation (repeated 3 times) as well as out-of-sample testing to validate our model performance. Another example is McDowell et al. (2021) who used GEMStat database to build a multiple linear regression model for predicting NOx—N values globally (for 7 years; centered around 2008). Multiple linear regression achieved *R*^2^ of 0.57, which is 36.7 % lower than our model performance.

Our proposed ML-driven model performance is also comparable with other existing studies that used process-based models to estimate dissolved inorganic nitrogen concentration which is mostly comprised of NOx. Examples include Green et al. (2004) (CTM, *R*^2^ = 0.52); Dumont et al. (2005) (NEWS-DIN, *R*^2^ = 0.78); Mayorga et al. (2010) (Global NEWS 2, *R*^2^ = 0.54); He et al. (2011a) (integrated TNCM, *R*^2^ = 0.81), McCrackin et al. (2014) (NEWS2-DIN-S, *R*^2^ = 0.80); Wang et al. (2020) (CAS-LSM, *R*^2^ = 0.62); and Huang et al. (2021) (integrated biogeochemical model, *R*^2^ = 0.84), who used far fewer sites to calibrate their models. This confirms the ability of our model in accurately establishing relationships between monthly NOx—N observations and predictor variables. It is worth mentioning that, given a high prediction accuracy achieved by our random forest model, we do not advocate ceasing development of the process-based models.

### 4.2 Physical justifiability of variable importance results

The random forest-based factor importance ranking and grouping results point at dominant anthropogenic and natural processes that govern the spatiotemporal variability in stream nitrogen concentrations globally. As illustrated in Fig. 6(b), model performance was highly sensitive to hydroclimatic variables, i.e., runoff, precipitation, and temperature, though runoff and precipitation tend to be more important than temperature (Fig. 6(a)). For example, in both the eastern and western USA, the flushing of *NO*_3_^−^ during snowmelt and spring storms is often considered as one of the largest sources of nitrogen pollution from many forested ecosystems (Cirmo and McDonnell, 1997). The high importance of these hydroclimatic variables also implies that climate change can strongly control nitrogen contamination across the globe. It is noteworthy that, with climate change expected to perturb hydroclimatic regimes in most regions across the world, climate change impacts on WQ have often been overshadowed by water quantity-related problems (i.e., droughts and floods).

Nitrogen loads, i.e., nitrogen in manure and fertilizer, also contributed the most to model accuracy (Fig. 6(b)). There is abundant evidence of increased nitrogen concentration in runoff from agricultural fields. The importance of this group can be justified because of both cropland expansion and raised fertilizer and manure application rates in per unit cropland area globally. The role of nitrogen in manure as one of the chief determinants of nitrogen level is also reflected in Fig. 6(a), wherein this variable is among the top four strongly influential predictors. Based on FAO (2018)’s estimates, more than 50 % of the global manure‑nitrogen input (both manure applied to soil and manure left on pasture) was produced by cattle in 2005. Moreover, stocks of pigs increased by ∼140 % worldwide, which caused more than five-fold increases in the nitrogen applied to soils from pig manure during 1961–2014 in Africa and Asia (FAO, 2018). This together with our modelling results highlight that livestock activities (e.g., manure/slurry application, animal housing, milk parlour washings, etc.) is one of the primary accelerators of water contamination in global scale. This finding supports the hotspot analysis results (Fig. 5) as well. Based on our analysis, some of the river basins, where nitrogen pollution is most severe, are in the top manure and fertilizer-consuming countries. For example, China, India, the US, Brazil, and Pakistan accounted for more than 60 % of global fertilizer consumption (Lu and Tian, 2017).

Our variable importance analysis shows that the group of predictors representing topographical features are among the moderately influential factors, with elevation being the most important variable (see Fig. 6(a) and (b)). In fact, topography is the key determinant of heat, water, and transport/redistribution of sediment and nutrients over the land surface, thereby influencing various process in nitrogen cycling, including erosion, water storage, nutrients leaching, and soil organic matter translocation.

It is worth mentioning that urban features proved less important to overall model accuracy, where wastewater production was the least influential predictor for NOx—N (see Fig. 6(a) and (b)), clearly because it is not the largest source of nitrogen contamination of surface water at global scale (Billen et al., 2013). It has been shown that discharges from urban areas and wastewater treatment plants are typically strong predictors for phosphorous exports rather than nitrogen (Castillo, 2010). However, the disposal of wastewater with a low level of treatment might have considerable implications for managing anthropogenic nitrogen flows in highly populated river basins, as the point sources of nitrogen are primarily associated with wastewater drainage. Note that the impact of wastewater treatment on model predictions was higher than wastewater production in Fig. 6(a).

### 4.3 Model uncertainty and caveat

Interpreting any data-driven modelling results indeed requires consideration of uncertainties, assumptions, and constraints of the analysis. In this sub-section, we describe various sources of uncertainty on global scale and explain potential limitations of our ML-driven approach. These uncertainties underscore the need for improved understanding of the interactions between nitrogen cycling and other long-term global changes in society, agriculture, and the environment.

We do recognize random forest has certain limitations. First, a lack of interpretability limits any random forest-based model's applicability because there is a set of hundreds of decision trees in the structure of the model. Second, the use of random forest needs caution when making predictions in an extrapolative manner, though our out-of-sample test showed an acceptable model performance.

Third, it has been often argued that the feature importance ranking measured by random forest might be sensitive to the relationships between variables and to the nature and scale of the predictor variable itself. Strobl et al. (2007) reported that when using permutation-based mean decrease in prediction accuracy as an importance measure, there might be bias in estimating importance of the correlated variables. Also, Pham et al. (2021) asserted that permutation-based metrics can underestimate importance of zero-inflated variables that are heavily skewed (e.g., cropland area) compared to variables that are more normally distributed. This might explain why the fraction of cropland area does not seem to have a significant contribution to our model performance (see Fig. 6(a)). We know that stream WQ is highly related to runoff from agricultural areas and fertilizer application during agricultural production. It is very surprising, however, to see such a low importance score for cropland area. A possible explanation is the correlation between agricultural fraction of land area and other variables. In other words, the impact of cropland area might be accounted for by the other correlated variable such as nitrogen in manure or topographical features.

The data- and scale-related uncertainties are noted as another important caveat. In this study, natural and anthropogenic predictors used for random forest model construction were collected from various databases. This can add uncertainties into the results due to the coarse resolution of some databases, and we acknowledge that upscaling is subject to considerable uncertainties. For example, the fraction of land responsible for most of the global nutrient application is likely to be overestimated here because of the coarse spatial resolution. Additionally, most of the predictor variables were derived from model-based estimates, which were designed to produce global-coverage data, and thus can have unavoidable uncertainty. Hence, more experimental studies are necessary for developing reliable datasets.

Lastly, sampling bias is a critical source of uncertainty in our work. Even though GEMStat provides observations of NOx—N from 718 stations at global scale, these monitoring stations are not well-distributed worldwide. By way of example, the distribution of stations is denser in Latin America, India, and western Europe, but there is a paucity of ground truth in Africa, central and north Asia (see Fig. 1). The resulting uncertainty may hinder our ability to thoroughly explore the underlying patterns and mechanisms controlling nitrogen dynamics globally, which should be addressed in future studies. Although our model achieved reasonable accuracy (*R*^*2*^ = 0.71) when making predictions on unseen data (but within the scope of the system), this ability is presumably limited to the new data if these are ‘*similar*’ to the training data. In fact, when using a narrow dataset as a baseline, most ML algorithms cannot perform well for data outside the training range. In situations such as predicting extreme events, predictions are highly uncertain due to the model's lack of knowledge about the new environmental properties which are significantly different from what has been observed in the training phase. To further investigate the generalizability of the trained ML models, using the ‘*area of applicability*’ approach (Meyer and Pebesma, 2021) together with recent developments in assessing prediction uncertainty (März, 2019) might be a promising starting point.

In supervised learning problems of environmental science, however, this is a common challenge as we typically encounter very small sample size that have gold-standard ground truth. It is often the case that observations used as training data for predictive modelling are not evenly distributed over study domain and predictions are subsequently made for areas that are suffering from lack of sufficient training data. In such circumstances, modelers assume that the learned mechanisms and relationships between predictors and responses are still valid, and thus the model can be transferred beyond the training space (e.g., to new geographic locations).

### 4.4 Future directions

Several key managerial implications can be drawn from this study. In particular, the proposed model can help assess the state of worldwide aquatic biodiversity, determine water-related health hazard over large areas, and evaluate impacts of global drivers such as climate on WQ. The spatial and temporal results derived from our proposed model can be used by ecological, hydrological, and human health models as well as by decision makers in two important directions. First, they can be applied in the context of scenario analysis to explore the nitrogen concentration's sensitivity to a wide range of plausible future changes (e.g., land use change). Second, they are particularly useful for informing global policy and governance debates concerning eutrophication or toxicity. Another fruitful area for further research is the application of the proposed model in integrated modelling and studies of interactions between various large-scale drivers of global nitrogen cycling that are difficult to account for in smaller scales, such as economy or international trade of food and animal feed.

Also, future work may include employing advanced global sensitivity and uncertainty analysis techniques for uncertainty apportionment. As suggested by Sheikholeslami and Razavi (2020) and Sheikholeslami et al. (2021), the given-data techniques are well-suited for this purpose when dealing with data-driven models. Furthermore, there are ample possibilities for development of physics-informed (or theory-guided) ML-driven WQ models to learn a wide range of physics relevant to hydrologic processes (Nearing et al., 2021). For example, an intriguing, simple strategy is that the performance metric used to train ML models can be modified to account for the physical consistency of the model predictions. Depending on the scale of spatial and temporal inference, we encourage use of hybrid models and model ensembles that integrate ML methods and process-based approach (see, e.g., Kraft et al., 2020).

## 5 Conclusions

The process-based models have typically struggled to fully represent the extremely complex biogeochemical processes and spatial variability that is evident in measured nitrogen concentrations. We addressed this challenge by building an ML-driven model that uses random forests algorithm and enables inference about the macro-scale behavior of the water quality (i.e., nitrate-nitrite concentration). Our analysis was global in scope, focusing on 520 major river basins. We found that 90 % of the variation in nitrate-nitrite concentrations is predictable from the selected covariates using the proposed random forest model. However, when we conducted out-of-sample validation to predict unseen data, we realized that only 71 % of the variability has been accounted for.

Our comprehensive analysis of the in-stream nitrogen patterns from 1990 to 2013 revealed hot spots of nitrogen pollution globally. On average, in United States, Europe, India, Pakistan, and China nitrogen pressures have occurred over large areas during 1990–2013, where severe water pollution occurs, depending on climate, drainage networks, and other factors. We also observed an increasing gradient of nitrogen concentration change from 1990s to 2000s in China, eastern and central parts of Canada, western United States, Baltic states, southern Finland, Pakistan, parts of Russia, mainland southeast Asia, and south-eastern Australia. In most of these regions, the high-volume and intensive crop and livestock production permitted by the increased fertilizer supplies has led to substantial increase in nitrogen flows during 1990–2013 (see, e.g., Zhang et al., 2022; Gao et al., 2020). Given the anticipated increase in human and livestock population, the losses of reactive nitrogen can trigger major environmental threats not only to water bodies but the air and soil with repercussions for human health and biodiversity.

Moreover, the utilized grouping-based variable importance measure helped us to identify relevant groups of predictors that are more important to achieve good model accuracy. These high importance groups lent insight into the dominant mechanisms underlying stream nitrogen concentration globally. Variable importance analysis confirmed the prominent role of diffuse non-point source nitrogen-inputs to surface waters from agriculture in nitrogen pollution of the river systems. Nutrient runoff from agriculture is typically associated with fertilizer use and livestock activity. Thus, implementing technical measures for improving crop-livestock farming practices must be at the forefront in reducing nitrogen environmental losses. The predicted nitrogen levels also showed a significant sensitivity to hydroclimatic variables, which will be of growing concern in the context of global climate change.

Finally, various sources of uncertainty in our analysis were described and potential limitations of the proposed ML-driven approach were identified. These uncertainties underscore the need for improved understanding of the interactions between nitrogen cycling and other long-term global changes in society, agriculture, and the environment (Lo Piano et al., 2022). Although we demonstrated the utility of the proposed random forest model in simulating nitrogen dynamics, the overall modelling procedure presented here is also well-suited to predicting other WQ indicators, such as phosphorus concentrations or salinity, particularly at large spatial scales and un-gauged river basins.

## CRediT authorship contribution statement

**Razi Sheikholeslami:** Conceptualization, Methodology, Software, Validation, Formal analysis, Investigation, Data curation, Writing- Original draft preparation, Writing- Reviewing and Editing, Visualization. **Jim W Hall:** Conceptualization, Investigation, Writing- Reviewing and Editing, Supervision, Funding acquisition.

## Declaration of competing interest

We declare no conflict of interest associated with this publication.

## Data Availability

The modelling was performed in R statistical computing environment, which provides multiple open-source packages for ML. In the spirit of reproducible research, the full procedure of the proposed global model, starting from the data collection and pre-processing to the 0.5-degree raster predictions will be made available on request to the corresponding author (R. Sheikholeslami, razi.sheikholeslami@sharif.edu).

## Appendix A Supplementary data and results

Supplementary material Image 1
