# Supplementary material for: Global patterns and key drivers of stream nitrogen concentration: A machine learning approach
Source: Sci Total Environ. 2023 Apr 10;868:161623. doi: 10.1016/j.scitotenv.2023.161623 (PMC10933795; doi:10.1016/j.scitotenv.2023.161623)
Supplement: Supplementary file 1 — Supplementary material [file mmc1.docx]

**Article Title:**

**Global Patterns and Key Drivers of Stream Nitrogen Concentration: A Machine Learning Approach**

Razi Sheikholeslami*^1,2,3^, Jim W. Hall^1,2^

^1^ School of Geography and the Environment, University of Oxford, Oxford, UK.

^2^ Environmental Change Institute, University of Oxford, Oxford, UK.

^3^ Department of Civil Engineering, Sharif University of Technology, Tehran, Iran.

# Corresponding author at: School of Geography and the Environment, University of Oxford, South Parks Road, OX13QY, Oxford, UK. Now at Department of Civil Engineering, Sharif University of Technology, Azadi Ave., P.O. Box 1458889694, Tehran, Iran. E-mail addresses: [razi.sheikholeslami@ouce.ox.ac.uk](mailto:razi.sheikholeslami@ouce.ox.ac.uk); [razi.sheikholeslami@sharif.edu](mailto:razi.sheikholeslami@sharif.edu) (R. Sheikholeslami)

**Funding Statement:** This research was funded by the Wellcome Trust, Our Planet Our Health (Livestock, Environment and People - LEAP), award number 205212/Z/16/Z.

**Disclosure Statement:** The authors declare no conflict of interest.

**Supplementary material**

The following supporting information is available for this article:

**Fig. A1** Map of major global river basins studied in this paper

**Fig. A2** Latitudinal distribution of NOx―N concentrations

**Table A1** An overview of predictor variables


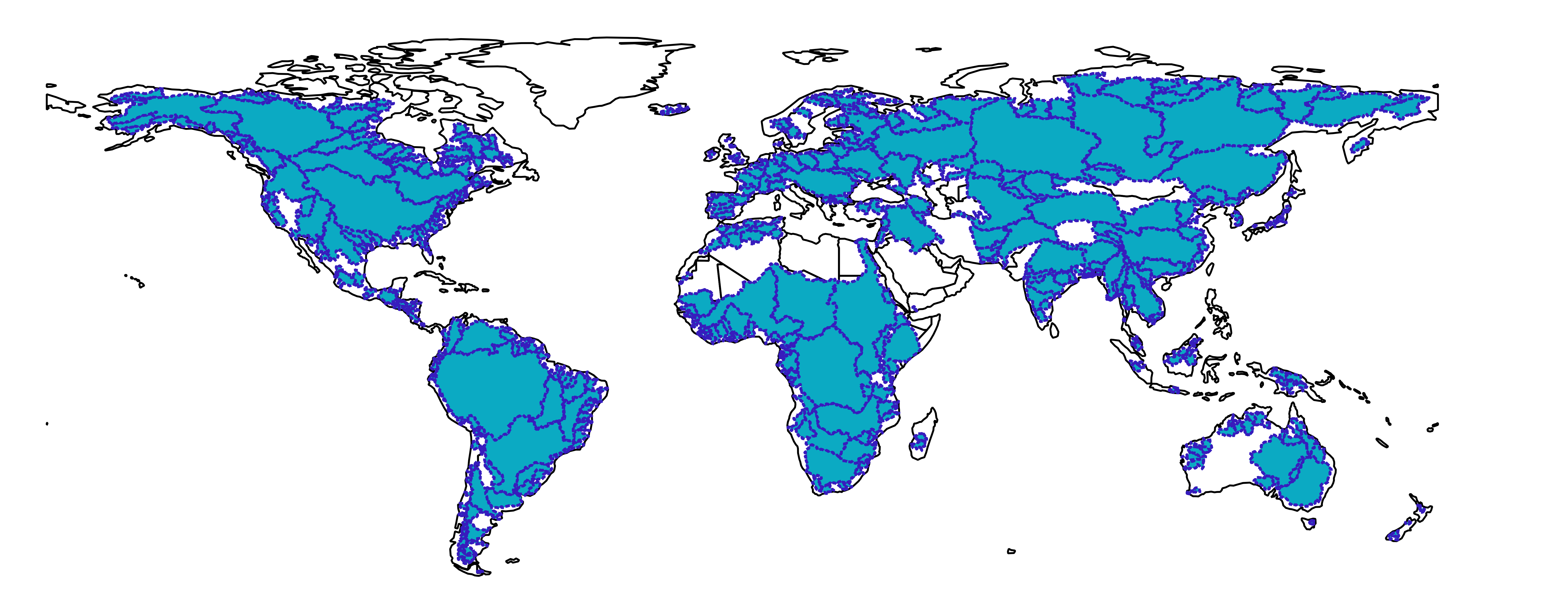


**Figure A1**. Map showing major global river basins studied in this paper. Shapes of 520 basins were extracted from the Global Runoff Data Centre (GRDC, 2020).


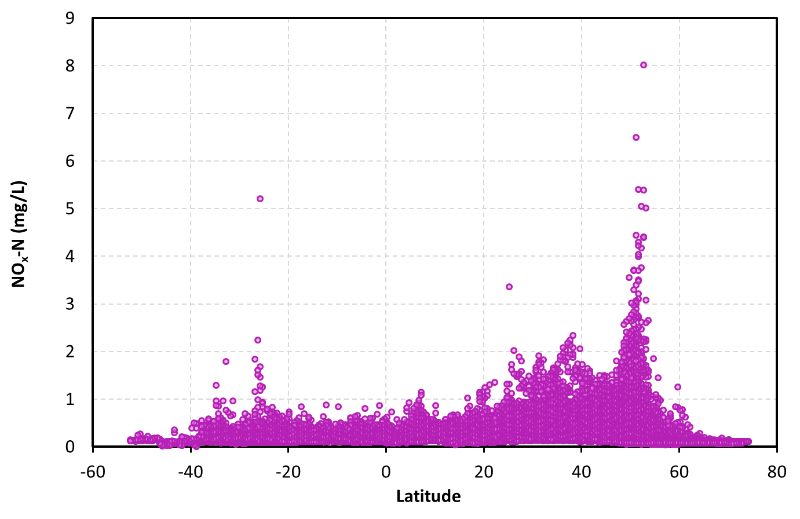


Figure A2. The summarized results along latitudinal gradients for NOx―N concentrations obtained from Fig. 5. Each pink dot represents the value of each 0.5° resolution grid cell.

**Table A1**. An overview of predictor variables, their spatiotemporal scales, and sources used in this study.

|  | Description | Spatial scale | | Temporal scale | | Source |
| --- | --- | --- | --- | --- | --- | --- |
| Variable |  | *Resolution* | *unit* | *Time step* | *coverage* |  |
| *Population* | Human population | 0.5-degree | number of people | Annual | 1990-2015 | Kummu et al. (2018) |
| *WWP* | Wastewater production | 5-arcmin | m^3^ yr^−1^ | - | 2015 | Jones et al. (2021) |
| *WWT* | Wastewater treatment | 5-arcmin | percentage | - | 2015 | Jones et al. (2021) |
| *Crop.frac* | Cropland fraction | 0.5-degree | percentage | Annual | 1961-2014 | Jackson et al. (2019) |
| *Urban.area* | Urban fraction | 1/8-degree | percentage | - | 2000 | Gao and O’Neill (2020) |
| *Forest.frac* | Forest cover fraction | 0.5-degree | percentage | Annual | 2000-2019 | Townsend and DiMiceli (2020) |
| *N.Manure* | Nitrogen in manure | 0.5-degree | Kg ha^−1^ | - | 2000 | Potter et al. (2010) |
| *N.fertlz* | Synthetic nitrogen fertilizer use | 0.5-degree | gN m^−2^ yr^−1^ | Annual | 1900-2013 | Lu and Tian (2017) |
| *Precipitation* | Precipitation | 0.5-degree | cm | Monthly | 1900-2014 | Willmott, and Matsuura (2001) |
| *Temperature* | Air temperature | 0.5-degree | °C | Monthly | 1900-2014 | Willmott and Matsuura (2001) |
| *Runoff* | Runoff | 0.5-degree | mm day^−1^ | Monthly | 1902-2014 | Ghiggi et al. (2019) |
| *Upstream.area* | Upstream area | 0.5-degree | km^2^ | - | 2011 | Wu et al. (2011) |
| *Elevation* | DEM | 15-arc-second | m | - | 2010 | Danielson and Gesch (2011) |

**References**

Danielson, J.J. and Gesch, D.B.: Global multi-resolution terrain elevation data 2010 (GMTED2010), In: Open-File Report. <https://doi.org/10.5066/F7J38R2N>, 2011.

Gao, J. and O’Neill, B.C.: Mapping global urban land for the 21st century with data-driven simulations and shared socioeconomic pathways. Nature communications, 11, 2302, <https://doi.org/10.1038/s41467-020-15788-7>, 2020.

Ghiggi, G., Humphrey, V., Seneviratne, S. I., and Gudmundsson, L.: GRUN: an observation-based global gridded runoff dataset from 1902 to 2014, Earth System Science Data, 11(4), 1655-1674, <https://doi.org/10.5194/essd-11-1655-2019>, 2019.

Jackson, N. D., Konar, M., Debaere, P., and Estes, L.: Probabilistic global maps of crop-specific areas from 1961 to 2014, Environmental Research Letters, 14(9), 094023, <https://doi.org/10.1088/1748-9326/ab3b93>, 2019.

Jones, E. R., van Vliet, M. T., Qadir, M., and Bierkens, M. F.: Country-level and gridded estimates of wastewater production, collection, treatment and reuse, Earth System Science Data, 13(2), 237-254, <https://doi.org/10.5194/essd-13-237-2021>, 2021.

Kummu, M., Taka, M., and Guillaume, J. H.: Gridded global datasets for gross domestic product and Human Development Index over 1990–2015, Scientific Data, 5, 180004, <https://doi.org/10.1038/sdata.2018.4>, 2018.

Lu, C. and Tian, H.: Global nitrogen and phosphorus fertilizer use for agriculture production in the past half century: shifted hot spots and nutrient imbalance, Earth System Science Data, 9(1), 181-192, <https://doi.org/10.5194/essd-9-181-2017>, 2017.

Potter, P., Ramankutty, N., Bennett, E. M., and Donner, S. D.: Characterizing the spatial patterns of global fertilizer application and manure production, Earth Interactions, 14(2), 1-22, <https://doi.org/10.1175/2009EI288.1>, 2010.

Townsend, J., and DiMiceli, C. - University of Maryland and MODAPS SIPS - NASA. MOD44B MODIS/Terra Vegetation Continuous Fields Yearly L3 Global 500m SIN Grid. NASA LP DAAC. <http://doi.org/10.5067/MODIS/MOD44B.006>, 2015.

Willmott, C. J. and K. Matsuura: Terrestrial air temperature and precipitation: monthly and annual time series (1900 - 2014), <https://psl.noaa.gov/data/gridded/data.UDel_AirT_Precip.html#detail>, 2001.

Wu, H., Kimball, J. S., Mantua, N., and Stanford, J. Automated upscaling of river networks for macroscale 1104 hydrological modeling, Water Resources Research, 47(3), W03517, <https://doi.org/10.1029/2009WR008871>, 2011.
